# Designing a Process for Cardiology Patient Transfers: A Quality Improvement, Descriptive Study on Interprovider Communication and Resident Education

**DOI:** 10.1097/pq9.0000000000000300

**Published:** 2020-05-18

**Authors:** John T. Kulesa, Sheri L. Balsara, Emanuel T. Ghebremariam, Jessica Colyer

**Affiliations:** From the Children’s National Medical Center, Washington, D.C.

## Abstract

**Background:**

Patient transitions create vulnerability for care teams. Failures in the handoff process result in communication errors and knowledge gaps, mainly when the handoff occurs between resident and expert-level subspecialty clinicians. The authors set out to develop a standardized handoff using resident comfort as a proxy for implementation. The primary measurable aim of this study was to increase the percentage of pediatric residents who self-reported comfort in assuming care of patients transitioned from the cardiac intensive care unit to the cardiology acute care unit.

**Methods:**

Investigators surveyed residents at a 323-bed pediatric hospital on their handoff experiences. The study team performed a Failure Mode Effect Analysis and created a key driver diagram. Interventions included a transfer checklist and algorithm, a huddle between care teams, and education surrounding the transfer process.

**Results:**

Residents completed a survey before (n = 74) or after (n = 23) intervention. The percentage of residents who reported feeling “always” or “very often” prepared to care for patients at the time of transfer increased from 15% to 83%. The percentage of residents who reported that they “always” or “very often” had concerns about floor appropriateness decreased from 23% to 4%.

**Conclusions:**

The authors designed a transfer process to improve communication, resident-level education, and psychological safety among team members to ensure safe, thorough handoffs between providers with different levels of training. Although we cannot definitively conclude that resident comfort improved due to a small “n” postintervention, we offer a description outlining process changes, barriers to implementation, and lessons learned.

## INTRODUCTION

Preventable adverse events are a significant source of patient morbidity and mortality in the hospital setting.^1,2^ Patient transitions create vulnerability for patients and care teams, as errors in communication during transitions increase the likelihood of adverse events.^3,4^ Implementation of a standardized process decreases handoff-related medical error and, therefore, the frequency of preventable adverse events.^5,6^ Standardization is associated with more comprehensive written handoffs, more controlled handoff environments, and increased time spent in direct contact with patients.^6^

Although the literature describes workflow patterns surrounding transfers from the intensive care unit (ICU) to the acute care unit,^7–9^ there is a lack of evidence to support a standardized physician handoff process in this setting. Furthermore, studies rarely describe the handoff process between providers of different levels of medical training. However, expert-level clinicians may not provide level-appropriate information to resident learners, and residents may avoid clarifying questions for fear of being seen as incompetent.^9–11^

The goal of this quality improvement (QI) initiative was to design a standardized transfer process for patients transitioning from the cardiac ICU (CICU) to the cardiology acute care unit (CU) to improve handoff communication for providers at different levels of training. Before this study, serious safety events occurred locally due to failures in the transfer process. The authors describe the iterative process by which we developed a standardized handoff using resident self-reported comfort as a proxy for implementation. The primary aim was to increase the percentage of pediatric residents who “always” or “very often” self-reported comfort in assuming care of newly transferred patients by 25% in 4 months. Although resident self-reported comfort is not equivalent to competency, it is a frequent surrogate.^12–14^ The study group defined competency as being adequately prepared to manage the patient at the time of transfer.

## METHODS

### Setting and Stakeholders

This study took place in a 323-bed academic urban free-standing children’s hospital. The CICU at this institution consists of 26 beds geographically isolated from the general pediatric ICU. Approximately 600 patients are admitted to the CICU annually. Over 300 patients transfer from the CICU to the cardiology service yearly.

At this hospital, patients requiring noninvasive positive-pressure ventilation chronically overnight are accepted to the CU if they remain on home settings. All mechanically ventilated, tracheostomy-dependent patients are managed in the ICU. Patients on stable continuous intravenous milrinone therapy are accepted, although other continuous inotropes are managed in the CICU.

CICU patients are cared for by a multidisciplinary team, including cardiac critical care attending physician and a critical care, neonatology, or cardiology fellow or nurse practitioner. Generally, CICU patients transfer to the cardiovascular surgery team or the cardiology team, composed of a cardiology attending physician, a cardiology fellow, and multiple second-year pediatric residents. The CICU rotation is the first rotation in which critical care fellows place orders and mediate transfers. Daytime transfers from the CICU to the CU were the focus of this work to pilot interventions with all stakeholders present.

The quality team consisted of 2 third-year residents, a performance improvement consultant, and the medical director of the cardiology unit and inpatient cardiology. Critical care attending physicians acted as champions for the project. This project was a QI initiative at our institution and not human subject research. As such, it did not require review and approval by the institutional review board.

### Transfer Process

Before this study, the CICU transfer process was not explicitly defined. CICU transfers lacked clear guidelines for when to initiate communication, what information should be conveyed, and what parties should be involved. The quality team interviewed residents and fellows, who outlined the following process when describing transfers that went well. The team later adopted this process as the gold standard for this hospital system.

Provider teams identify patients as stable for transfer when they determine, with reasonable clinical confidence, that the patient does not require support beyond the usual capabilities of the CU. When stable, 2 handoffs occur, typically over the phone. The first handoff, limited to active cardiology problems, involves the CU fellow or attending and the CICU fellow or attending. If appropriate, the cardiologist accepts the patient at this time, and the CICU nursing staff communicates with administrative staff to request a bed.

When a CU bed is assigned, the CICU primary provider delivers a comprehensive system-based verbal sign-out to the cardiology resident. Because this second handoff may occur hours after the first, depending on bed availability, the CICU team conveys any recent changes in clinical status. The accepting resident assesses the patient in the ICU and places the transfer order within 1 hour of bed availability. If there are resident-level concerns, residents initiate attending-to-attending communication. The ICU team provides written documentation of the hospital course to complete the transfer process. The resident then staffs the patient with the CU fellow.

### Identifying the Problem

**Fig. 1.**
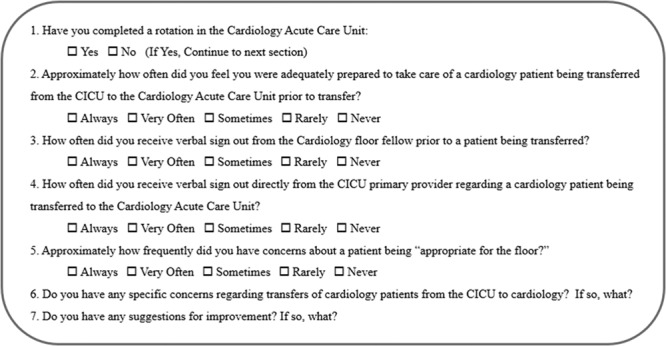
Preintervention survey.

The quality team distributed an anonymous electronic survey to the pediatric residency program (Fig. 1). The survey probed residents on their experiences surrounding the transfer process.

Respondents felt ill-equipped to care for complex cardiology patients. They cited situations in which it was challenging to identify and manage acute decompensation in this patient population. Although there is direct oversight by an in-house cardiology fellow, residents felt unsure of when to escalate.

Survey participants also reported that the fellow-to-resident handoff was often limited to the cardiovascular system. Inadequate information resulted in medical errors and near-miss events. For example, 1 patient had a severe allergic reaction to a medication, and after transferring the patient to the cardiology service, the resident ordered the same medicine again. The patient’s family notified the medical team of the previous reaction. Another patient with chronic pain on home opiates temporarily required an escalation in pain management in the ICU setting. Dosing was not readjusted at the time of transfer, and the patient required naloxone to reverse narcotic-associated hypoventilation. Written documentation, used to fill gaps in communication, was often incomplete or untimely.

Survey respondents also felt excluded from the transfer process. Residents attributed this to inadequate fellow education surrounding transfer logistics. For example, CICU fellows placed transfer orders, and patients were transported without resident notification, posing a particular risk during resident transitions and at times of a high census.

**Fig. 2.**
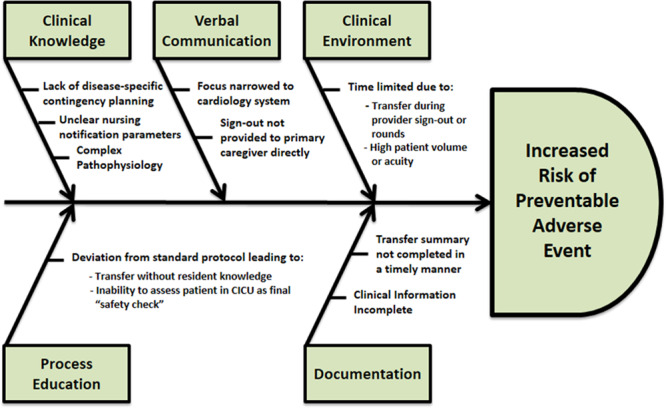
Fishbone diagram.

**Fig. 3.**
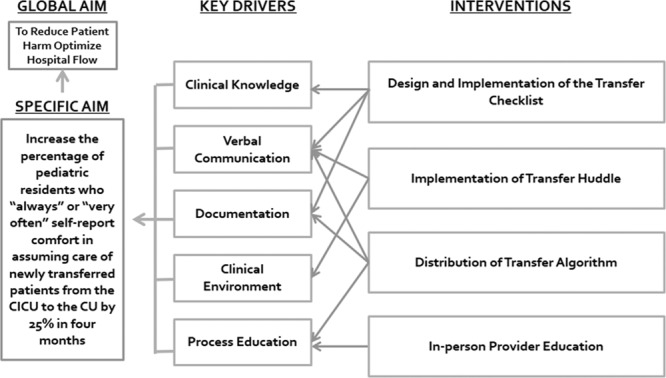
Key driver diagram.

Informed by survey results, the quality team performed a failure mode effect analysis and created a fishbone diagram (Fig. 2) and key driver diagram (Fig. 3). The team designed and modified interventions throughout several plan-do-study-act cycles.

### Interventions and Implementation Strategy

#### Transfer Checklist

The quality team designed a transfer checklist to be completed by the cardiology resident. Each month, the team provided orientation to the residents and explained each checklist item. Although the team designed the checklist as a process checklist to monitor and communicate deficiencies with stakeholders on an ongoing basis, we informed residents that the checklist order was not rigid. The team made blank checklists available in a folder in the cardiology team room and asked residents to complete one checklist for each patient transferred. The items listed in Table 1, except for “Verbal sign-out received from CICU and/or CU fellow,” were the items present on the checklist provided to residents.

**Table 1.**
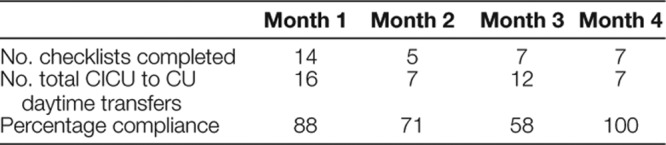
Transfer Checklist Compliance: Number of Checklists Completed and Percentage Compliance

There were several motivators guiding checklist design. First, the team hoped to define the components of a complete transfer. Backed by critical care physician leadership, the checklist substantiated resident requests for process adherence. The team notified residents that “Verbal sign-out received from CU fellow” was included as a checklist item solely for data collection, and that the team expected residents to speak with the primary ICU provider.

Second, the team designed checklist items to encourage information gathering and resident education. For example, while orienting residents on the meaning of “Discussion of plan and floor appropriateness with CU fellow,” the team instructed residents to ask questions about the patient’s underlying pathology or disease state, an acceptable baseline, disease-specific signs of decompensation, management of unfamiliar or high-risk interventions, and appropriate contingency plans. The goal of this checklist item was to empower residents to identify their knowledge gaps and address them with the accepting fellow, and to engender a culture where residents felt comfortable advocating for improved communication and normalizing the experience of unfamiliarity and the need to ask multiple questions.

The quality team included “Discussion of plan and floor appropriateness with nursing staff” to encourage closed-loop communication, help residents define notification parameters, and address CU nursing concerns. At our institution, nurses are often familiar with hospital policies surrounding interventions typically managed in the ICU. The team encouraged nurses to communicate their usual scope of practice. With this insight, residents crafted more robust questions for the fellow.

The team iteratively modified the checklist during several plan-do-study-act cycles. First, team members performed biweekly in-person “check-ins” to encourage checklist adherence and discuss how residents were using the checklist and why. Second, the team defined a consistent location for the checklist folder and added the checklist to the electronic handoff document. Third, the team condensed checklist items to reduce perceived workload and added a checklist item for residents to describe situational barriers to the handoff process.

#### Transfer Algorithm

**Fig. 4.**
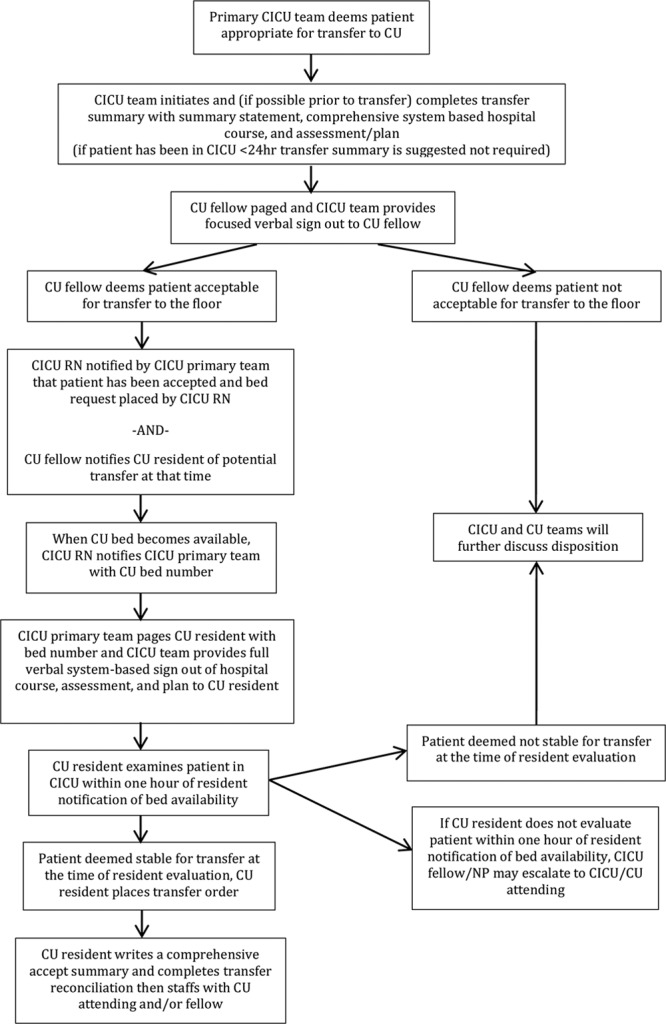
CICU to CU transfer algorithm. RN, Registered Nurse; NP, Nurse Practitioner.

The quality team created a transfer algorithm (Fig. 4) to describe the transfer process. Although the overall structure distills to 2 handoffs, one of which is a partial handoff limited to the cardiology system, the team chose to include details on administrative tasks. The team reviewed the algorithm in-person at a critical care fellow administrative meeting 3 months into the study period and posted the algorithm in physician workrooms.

Although extending in-person education to the entire CICU staff was not feasible due to variable work schedules, the team emailed the algorithm to CICU primary providers, including fellows and nurse practitioners. Two critical care attendings emailed CICU primary providers supporting the procedures outlined. The team encouraged residents to provide education and reference to the posted algorithm during the transfer process. The team provided targeted intermittent feedback based on checklist compliance.

#### Daily Transfer Huddle

The quality team designed a daily transfer huddle between the CICU and cardiology attendings, fellows, and residents as a forum to identify potential transfers before morning rounds. During the huddle, the CICU provided a unified handoff, limited to the cardiology system, to members of the accepting team. Early identification of transfers allowed residents to evaluate and accumulate information on patients before rounds. The huddle also served as a medium to promote education on the transfer process as needed.

This intervention, however, proved ineffective. During implementation, both the CICU and CU teams changed the start time of rounds. Huddles frequently interfered with learning and patient care activities and initially occurred during morning reports, which limited resident participation. ICU patient acuity influenced CICU fellow availability and representation. The teams trialed transfer huddles for roughly 3 weeks, 4 or fewer attempts each week, with variable start time and resident participation. We discontinued transfer huddles due to these logistic constraints.

### Evaluation Plan

This study followed the Donabedian model for QI. The team redistributed the preintervention survey 4 months into the study period and then performed a “spot check” 18 months after implementation to augment postintervention data and assess sustainability. Residents estimated how often they felt prepared to care for patients at the time of transfer and how often they had concerns regarding clinical status, recorded as outcome measures. The team monitored the fidelity of the transfer checklist intervention by tracking the completion of each checklist item and the number of completed checklists relative to the number of patients transferred, recorded as process measures. The team used the electronic medical record to review CICU “bounce-backs” within 48 hours of transfer and “lag time,” the time between transfer order placement and patient arrival on the CU, recorded as balancing measures.

## RESULTS

Before interventions, 63% (n = 74) of 118 residents completed the preintervention survey. A total of 70% (n = 52) of respondents completed the cardiology rotation before survey distribution and completed the entirety of the survey. A total of 75% (n = 8) of residents who completed the rotation postintervention responded to a secondary survey distributed after the 4-month study period, and 75% (n = 15) responded to a spot check survey 18 months after implementation.

On pre- and postintervention surveys, the percentage of residents who reported feeling “always” or “very often” prepared to care for patients at the time of transfer increased from 15% to 83% (88% at 4 months; 80% at 18 months). The percentage who reported “always” or “very often” having concerns about floor appropriateness decreased from 23% to 4% (0% at 4 months; 7% at 18 months).

**Fig. 5.**
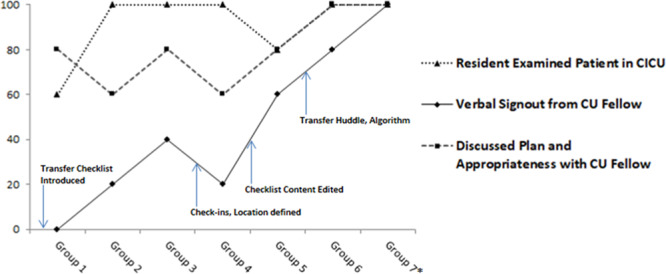
Compliance for select checklist items. *Group 7 included 3 patients, all other groups included 5 patients.

Table 1 includes the number of checklists completed each month and percentage compliance. Table 2 includes the percentage compliance for each checklist item within completed checklists, organized by groupings of every 5 patients transferred. Note that the final group consisted of 3 patients—Figure 5 displays compliance for 3 essential checklist items critical to the primary aim.

**Table 2.**
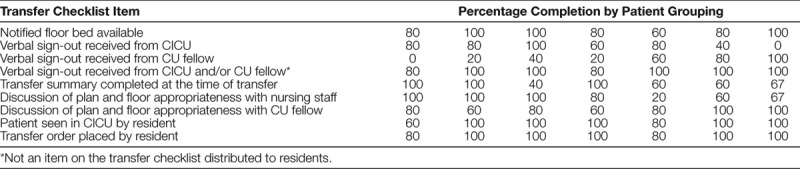
Transfer Checklist Compliance: Percentage Compliance for Each Checklist Item

There were several notable trends. As the study period progressed, residents received verbal sign-out from the CU fellow more frequently. Direct CICU-to-resident sign-out and discussion with the nursing staff before transfer declined. Overall, compliance with checklist completion varied.

In the 8 months directly before the first QI intervention, the median lag time was 78 minutes (interquartile range, 37–149 minutes), and 2 of 217 cardiology patient transfers resulted in unplanned bounce-backs. During the 4 months of the study period, the median lag time was 90 minutes (interquartile range, 56–144 minutes), and 1 of 97 cardiology patient transfers resulted in an unplanned bounce-back. No bounce-backs resulted from preventable adverse events.

Notably, the study duration postintervention was limited to 4 months due to environmental factors. The CU underwent renovations at that time, and the acute care team relocated. Checklists completed after the 4-month intervention period were unintentionally discarded in the moving process.

## DISCUSSION

The quality team designed a transfer process to ensure safe, thorough handoffs between providers with different levels of training. The team describes process changes implemented to optimize communication, resident-level education, and psychological safety between team members.

With regard to the checklist, items designed as markers for process compliance, such as “Transfer order placed by resident,” improved. The transfer checklist promoted education for CICU primary providers and decreased premature transfer orders. Attending-level communication increased buy-in. Increased frequency of “Verbal sign-out received from CU fellow” was an unintended outcome of this project. However, residents reported that learning increased as communication with the CU fellow improved. Residents attributed this trend to reliable access to the CU fellow, resident comfort with the established relationship, and the desire to sign-out in person rather than over the phone. Using survey data to uncover areas of vulnerability in the subspecialist-to-resident handoff, as reported by the receiving resident, and informing subspecialists of that data, improved the educational process.

Concerning the transfer algorithm, markers of process compliance, as above, grossly improved with transfer education. Stakeholder education strategies included in-person training at a critical care administrative meeting, email communication promoted by critical care leadership, and distribution of the algorithm posted in the CICU and cardiology team rooms. The quality team asked residents to encourage deliberate practice with the transfer algorithm. Residents provided in-action feedback to the transferring provider as needed. Reportedly, if residents did not reinforce the process, compliance decreased. Ideally, in-person education would occur for all CICU team members, but this was not feasible due to variable work hours.

The authors noted several differences between observed and anticipated outcomes. With increased CU fellow-to-resident handoffs, direct CICU handoffs decreased, although CU fellows more frequently received full system-based sign-out from the CICU when they were responsible for distributing this information to residents. Residents did not find value in seeking nursing input on patient acuity before accepting the patient. The CICU often finalized transfer summaries after the transfer; although with improved verbal communication, residents considered timely documentation less critical.

Checklist compliance fluctuated over time. With reliance on reporter data, barriers were likely multifactorial. First, motivation varied among residents, although anecdotal evidence from previous safety events increased resident incentive and biweekly check-ins reinforced learner expectancies. Second, if article checklists were not readily available at the time of transfer, particularly during rounds, residents were unlikely to disrupt their workflow to retrieve blank checklists. The quality team defined a set location for blank checklists and then attached the transfer checklist to the cardiology service electronic handoff document with resultant improvement.

During the study period, there was an incident during which a resident refused a patient because the CICU had not completed the transfer summary. This refusal delayed the patient transition during a time of high acuity and complicated a CICU admission from the operating room. During subsequent orientations, the quality team explicitly requested that residents avoid “hard stops” in the transfer process as a means to facilitate CICU reengagement.

### Limitations

We describe the limitations of a resident-run project with diverse stakeholders reliant on manual data collection. Limitations included a short study period, fragmented postintervention data, and a small “n” postintervention. The authors, therefore, cannot definitively conclude that resident comfort improved or establish a causal link between our interventions and resident comfort. Compliance fluctuated over time. Of the interventions, transfer huddles were least effective, and the quality team discontinued this intervention. This study took place at a large urban children’s hospital, so the results may not be generalizable to other contexts. Unplanned bounce-backs to the CICU were rare before and after interventions, so the impact is inconclusive. Median lag time increased slightly during the study period, although it is difficult to assess if this increase is attributable to the interventions.

## CONCLUSIONS

The study group describes one approach to uncover and address errors in interprovider communication during transfer and enhance resident education. This work serves as a pilot study for future QI initiatives surrounding subspecialty ICU to acute care unit transfers at this institution or in other contexts. Further analysis is needed to evaluate the interventions’ direct impact on patient safety and resident comfort; to ensure these processes do not negatively impact patient flow; to elicit the fellow, nursing, and attending perspective; and to assess sustainability further.

## DISCLOSURE

The authors have no financial interest to declare in relation to the content of this article.
